# Asymmetrical Deterministic Lateral Displacement Gaps for Dual Functions of Enhanced Separation and Throughput of Red Blood Cells

**DOI:** 10.1038/srep22934

**Published:** 2016-03-10

**Authors:** Kerwin Kwek Zeming, Thoriq Salafi, Chia-Hung Chen, Yong Zhang

**Affiliations:** 1 Department of Biomedical Engineering, National University of Singapore, 4 Engineering Drive 3, Block E4 #04-08, 117583, Singapore; 2 Cellular and Molecular Bioengineering Lab, National University of Singapore, Block E3A, #07-06, 7 Engineering Drive 1, 117574, Singapore; 3 Singapore Institute for Neurotechnology (SINAPSE), 28 Medical Dr. #05-COR, 117456, Singapore; 4 NUS Graduate School for Integrative Sciences and Engineering, Centre for Life Sciences (CeLS), National University of Singapore, 05-01 28 Medical Drive, Singapore 117456, Singapore

## Abstract

Deterministic lateral displacement (DLD) method for particle separation in microfluidic devices has been extensively used for particle separation in recent years due to its high resolution and robust separation. DLD has shown versatility for a wide spectrum of applications for sorting of micro particles such as parasites, blood cells to bacteria and DNA. DLD model is designed for spherical particles and efficient separation of blood cells is challenging due to non-uniform shape and size. Moreover, separation in sub-micron regime requires the gap size of DLD systems to be reduced which exponentially increases the device resistance, resulting in greatly reduced throughput. This paper shows how simple application of asymmetrical DLD gap-size by changing the ratio of lateral-gap (G_L_) to downstream-gap (G_D_) enables efficient separation of RBCs without greatly restricting throughput. This method reduces the need for challenging fabrication of DLD pillars and provides new insight to the current DLD model. The separation shows an increase in DLD critical diameter resolution (separate smaller particles) and increase selectivity for non-spherical RBCs. The RBCs separate better as compared to standard DLD model with symmetrical gap sizes. This method can be applied to separate non-spherical bacteria or sub-micron particles to enhance throughput and DLD resolution.

Deterministic lateral displacement (DLD) method for particle separation in microfluidic devices has been extensively used for particle separation in recent years since it was first published by Huang *et al*.^1^,^2^ Due to its high resolution and robust separation, DLD has shown versatility for a wide spectrum of applications for sorting of microparticles such as parasites^3^, blood cells^4^,^5^,^6^,^7^, circulating tumor cells^8^,^9^, bacteria^7^,^10^, spores^11^, and more recently, nanoparticle separation^12^ and DNA isolation^13^,^14^. While the DLD empirical model is well established in current research, its scope is restricted to spherical particles, cylindrical pillars and uniform gap-size across all adjacent pillars^15^. Thus, it has been challenging to separate non-spherical particles such as red blood cells (RBCs), bacteria and DNA. Many groups have worked on changing pillar shapes and device gaps to effectively separate these particles^16^,^17^. However, this will greatly increase fabrication complexity and restrict throughput by reducing gap-sizes in a DLD pillar array.

The DLD method uses rhombic or rotated square pillar arrays to redirect fluid laminar flow streams and each array arrangement would have a distinctive critical separating diameter. Particle larger than the critical cut-off diameter (D_c_) will be displaced laterally from its flow from the sample streamline while smaller particles flow unhindered in the pillar array by flowing within the fluid streamlines. Based on current DLD empirical model, DLD separation cut-off size and resolution depends on the array rotation angle or slant and lateral gap-size between pillars^6^:


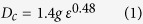


Where *D*_*c*_ is the critical diameter of particle for separation, *g* is the lateral gap or pore-size between pillars and *ε* is the slope of pillar array (tan *θ* = *ε*) when *θ* is the angle of gradient. DLD separation resolution depends on the lateral gap and row shift fraction of the pillar arrays. The resolution can be enhanced by reducing the lateral gap or gradient. However, smaller lateral gap size significantly amplifies the resistance of the array exponentially while lowering the gradient increases the length of the device significantly. The requirement of small gap for acquiring small critical diameter implies that submicron separation is challenging due to high flow resistance which drastically reduce the throughput for particles. Recent DLD works showed DNA separation with 1.7 μm gap flowed at rate of only 0.01 μl/min^14^. Furthermore, small gap size also imposes difficulty in submicron lithography fabrication. These challenges pose a limit for the advancement of DLD to separate submicron size particles.

DLD device design currently has same lateral and downstream gap size adjacent to the pillars and the DLD model states that the cross-sectional fluid flow profile of the lateral gap across the adjacent pillars critically determines the separation. However, in our research we found that varying the downstream gap-size can also enhance the particle separation. We also observed that current works on DLD is not consistent as some of them define the pillar gradient as *θ* to calculate the critical diameter, while others use row shift fraction 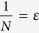. Most DLD research uses *ε* = *tanθ* which we found only true if the lateral and downstream gaps are of equal size^2^. This poses a challenge when the gaps within DLD device are asymmetrical as the estimation of D_c_ would be incorrect. Davis *et al*. was the first to separated blood components using asymmetrical gap sizes but only reported the use of lateral gap size for calculation of D_c_^6^. The calculated D_c_ of 6.6 μm is even larger than the downstream gap of 6 μm. The separation resembles more of cross-flow filtration rather than DLD since the size of white blood cells (>9 μm) are much larger than the downstream gap (6 μm). Moreover, the use and effects of asymmetrical gap sizes were not mentioned and discussed. The effect of varying downstream and lateral gaps in DLD currently has not been investigated. Thus, in this study we examined the effects of both lateral and downstream DLD gap size in DLD.

We hypothesized that asymmetrical DLD gaps performs two roles: lateral gap influences DLD throughput while the downstream gap enhances the DLD critical diameter (see Fig. 1). This dual functions of asymmetrical DLD gap potentially enhance particle separation without compromising the throughput respectively. Our findings bring new insight to the current DLD model and open new possibilities to enhance DLD separation. The tuning of downstream instead of lateral gap will be crucial for submicron separation in DLD to achieve the same critical diameter without reducing throughput. Thus this paper shows a different approach towards enhancing separation of RBCs and smaller sub-micron particles.

## Theory

### DLD Theory

In deterministic lateral displacement device, there are pillar arrays obstacle with specific gaps which allow the formation of several streamline flow between the gaps that separate particles to different direction depends on its size, deformation and shape. The distance between each pillar is set to have period of *λ*, gap *G* = *λ* − *D*_*p*_, and shifted row post of Δ*λ* = *ελ* with *ε* be the row shift fraction. DLD theory states that the number of flow stream that carries equal flux (N) can be determined by the following formula^1^.


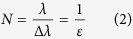


where N also depicts the number of pillar rows needed to shift to the next column. Current DLD theory states that the row shift fraction is also equal to the tangent of the tilted pillar row angle *θ*^2^.


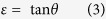


while most users design DLD device by applying this formula, we found out that 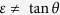 unless the lateral gap is equal to the downstream gap. The *ε* depends on lateral gap *G*_*L*_, downstream gap *G*_*D*_, pillar diameter *D*_*p*_ and tilt angle *θ*. The revised formula of *ε* which takes the downstream gap size and diameter into account is


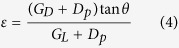


this newly revised formula shows that now for assymetrical flow, pillar diameter and gap size of both lateral and downstream play a role in determining the D_c_ from Eq. 1. If there is particle flowed through these array post with diameter larger than the critical particle size (*D*_*c*_), the particle will collide with the post and move towards the next streamline which is referred as bump mode flow. whereas the particle smaller than the critical particle size (*D*_*c*_) will move in zigzag mode and therefore follow the streamline^15^.

It is a common assumption that the cut off diameter of particle between the zigzag mode and bump mode, *D*_*c*_, or critical diameter size for separation is twice the width of the first streamline, *β*


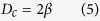


where it can be further approximated to the following equation for non-uniform velocity profile


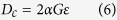


with *α* is unit-less number to correct for a non-uniform flow profile. After several characterization experiments with different gap size and *ε*, Davis *et al*. proposed an empirical formula (Equation 1) for critical diameter size.

### DLD Array Resistance

#### Systemic resistance of DLD device due to lateral gap (G_L_)

DLD array resistance calculation is crucial for designing a cell separation device as the throughput depends on the entire microfluidics array resistance. The resistance of the fluid flow in a microfluidics device can be calculated by the ratio of pressure difference (Δ*P*) to the flow rate (Q)


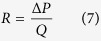


For a simple rectangular channel, the resistance can be derived with the assumption of gap less than depth (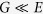) which result in the following theoretical equation^18^


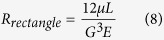


In which *μ* is viscosity and L is length. Davis *et al*. determined the DLD circle array resistance with the gap size equal to the post size and shift fraction of 0.1 through simulation of flow in COMSOL and found out the resistance of the circle post array can be computed by the following equation^5^.


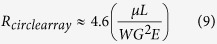


where W is the width of the arrays. From this derivation, the lateral gap of the circle DLD arrays contributes largest as it goes in square inverse to the resistance of the arrays.

#### Anisotropic resistance of adjacent DLD columns due to downstream gap (G_D_)

The changes in downstream gap has an expected effect on flow resistance between adjacent DLD columns. As the G_D_ reduces, the lateral resistance increases and the net flow of the fluid is in the direction of the DLD gradient. Thus we would expect increase anisotropic resistance between adjacent pillar columns which may influence the D_c_ of DLD. Lubbersen *et al*. have accounted for this additional fluid force which drives particle preferentially along the gradient of the sparse array^19^.

## Materials and Methods

### Computational Modeling parameters

To evaluate the effect of lateral and downstream gap on the array resistance, the relationship between the gap and resistance of the device is calculated computationally with COMSOL Multiphysics. We fixed the pressure drop and used the flow rate result from the 2 D flow simulation to compute the resistance value. The notation to determine the different asymmetrical gap ratios will be denoted in G_L_: G_D_ where G_L_ and G_D_ represent the lateral gap and downstream gap in μm respectively. The resistance of varying G_D_ of 15:2.5, 15:5, 15:7.5, 15:10 and 15:12.5 and varying G_L_ of 2.5:15, 5:15, 7.5:15, 10:15 and 12.5:15 were calculated with a fixed pillar diameter of 15μm, fixed viscosity, width and length of the channels, while the *ε* value used for the simulation was acquired from revised formula (Eq. 4) of row shift fraction. The obtained resistance values were then analyzed to see the relationship of gap size to the resistance of the arrays. The details of the simulation parameters can be found in the Supplementary Table-S1.

### Device design

To test the effects of asymmetrical DLD gap size on the D_c_ of the DLD device, we designed three DLD device with 9*μm*:9*μm*, 9*μm*:4*μm*, and 4*μm*:9*μm* gap sizes with pillar diameter of 9*μm* (Fig. 2). The devices have three input regions for buffer streams to sandwich the sample fluid stream and 22 output sub-channels to characterize the separation shown in Fig. 2(b). The input channels have filters and resistive pillars to balance the input pressures and ensure input flow stabilization. The sample exits the DLD pillars into 22 sub-channels which converge into a single output channel to allow a stable negative pressure driven flow (Fig. 2(d).). The designs were printed on to a chrome glass mask (IGI, Singapore) where the patterns were transferred to a silicon wafer using standard positive resist (AZ5214E, AZ Chemicals) lithography. A mask aligner (SUSS MA8) was used to transfer the patterns. The final device was etched using deep reactive ion etching (Oxford 180 DRIE) of 12 cycles to a depth of 11 μm. The wafer was cleaned using Piranha solution (Sigma Aldrich, Singapore) and a thin PDMS layer was used to cover the channels. 1 mm holes were punched for both the input reservoir and output negative pressure tubing port. The mask designs for the DLD devices can be found in Supplementary Fig. S1.

### Sample and Buffer Solutions

The particles used in this study were NIST standard polystyrene beads (Bangs Laboratories, USA) ranging from 1.5 μm, 2.0 μm, 2.5 μm and 3.0 μm. The beads were diluted in 0.01% pluronic F127 (Sigma Aldrich, Singapore) mixed in an ultrasonic bath for 5 min to ensure maximum monodispersity. Approximately 10 μl of RBC samples were extracted from a finger prick and were suspended in 1x Phosphate buffered saline solution (Life Technologies, Singapore) in a ratio of 1:10.

### Experimental method

The system was driven by a negative pressure at the outlet using retraction mode of a syringe pump, whereas the sample and buffer inlets are open-air reservoirs (Fig. 2). Surface treatment of the devices was performed by applying a surface coating of (tridecafluoro-1,1,2,2-tetrahydrooctyl)-1-trichlorosilane (Sigma, Singapore) via chemical vapor deposition in a vaccum desicator and a pre-flow treatment of 1% (w/v) Pluronic F-127 solution to prevent the attachment of bio-particles to the device surface. After 30 min of Pluronic treatment, the device was washed by loading fresh ultrapure DI water (Milipore 18 ohms DI water) into the reservoirs and flushed for 30 min. To drive the flow process, a 100 μl gas-tight Hamilton glass syringe was attached to a 150 μm diameter tube, which was inserted into the device outlet. A syringe pump (Chemyx) was attached to the syringe and the syringe piston was retracted at a flow rate of 0.5 μl/min. This flow rate was fixed throughout to ensure no fluctuations of pressure during the experiements. A pipette was used to load or wash the reservoirs with sample or buffer solutions. Sample loading takes less than 5 mins for flow stablization and washing steps would take no longer than 5 mins for the device to be visually cleared of previous sample.

To capture the separated beads and RBCs at the output of the DLD device, a high-speed Phantom camera (M310) was attached to a bright-field upright microscope at a magnification of 400x. Images of the particles at the output positions were captured from each video ranging from frame rates of 400 fps–2000 fps, and the beads at the respective output were counted manually from these video. A composite image of the video was made by layering 15 image sequence followed by image processing to increase contrast of the sample.

## Results and Discussion

### Asymmetrical gap-size DLD ratio

**Figure 1:**
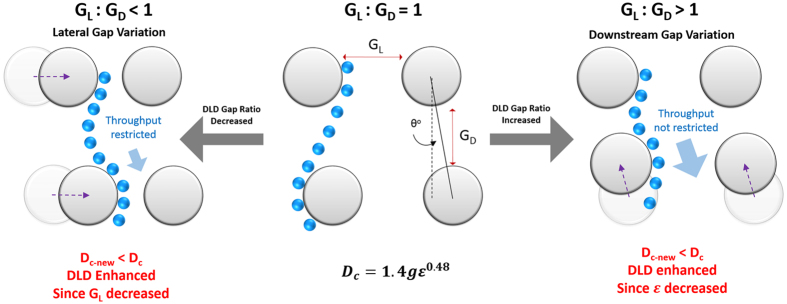
Schematics of asymmetric DLD gap-size ratio and its effects on throughput and D_c_ enhancement. The schematics depict two scenarios with different gap size ratios. One which has a lower DLD gap size ratio (G_L_ : G_D_ < 1) shows decrease in throughput and the other shows increase in throughput with a corresponding increase in gap size ratio (G_L_ : G_D_ > 1). Both cases, DLD is enhanced depicted by the path of the blue colour bead.

In this work, we explore the asymmetrical gap size ratio of DLD for the enhancement of particle separation and throughput (Fig. 1). The critical separation of DLD devices are determined by Eq. 1 which is governed by the lateral gap size (g) and the pillar row shift fraction *ε* which can be determined by Eqs 2 and 3. As the DLD gap size ratio decreases due to constriction of lateral gap, there will be an exponentially increasing fluid resistance due to the pillar array and thus the throughput will be restricted. However, the D_c_ will decrease and smaller particles will be able to be separated. This is because the lateral gap-size is reduced and based on Eq. 1 it should increase the resolution of separation by decreasing D_c_.

When the downstream gap is reduced, throughput of the DLD device will not be reduced drastically as the pillar forms a relatively wide channel (as oppose to gap ratio G_L_ : G_D_ less than 1). The Dc is also enhanced as the row shift fraction, *ε* is reduced. The row shift fraction in this case has to use Eq. 4 to be calculated. This is because the angle of gradient does not change, only the downstream gap changes. Hence, we hypothesize that decreasing G_D_ is favorable for DLD separation as it does not restrict throughput while at the same time enhance the particle separation.

### The effects of G_L_ and G_D_ on throughput of DLD device

We conducted flow simulation of DLD arrays and compared the effects of G_L_ and G_D_ gap size variation on array resistance using COMSOL Multiphysics. The *ε* value of different G_L_ and G_D_ were calculated from the revised *ε* formula (See Supplementary Table-S2). By normalizing the resistances of the varying gap sizes of G_L_ and G_D_ to the resistance of symmetric 15:15 DLD array reference, we were able to compare the relative resistance of varying G_L_ and G_D_ shown in Fig. 3(a). The normalized gap ratios were calculated by dividing the respective G_L_ or G_D_ (2.5, 5, 7.5, 10, 12.5 and 15 μm) with the reference gap-size of 15 μm. At a normalized gap of 0.167, the reduced G_L_ of 2.5:15 resulted in a 41 fold increase in resistance as compared to a 1.3 fold increase using a 15:2.5 pillar array. The results also confirm the inverse square influence of the G_L_ from Eq. 9. Interestingly, reducing G_D_ relative to the standard DLD array (G_L_ = G_D_) resulted in a proportional increase in resistance (see Supplementary Fig. S2).

Figure 3(b–d) shows the throughput between an array with 15:15, 15:2 and 2:15 gap sizes using their own color data range in the units of m/s. The array of 15:15 has the highest throughput, followed by 15:2 gap size which shows significantly higher flow velocity profile compared to the low velocity profile on 2:15 gap size. The higher throughput implies that the reduction in downstream gap is preferentially advantageous for separation of small critical diameter particles in sub-micron regime where DLD device resistance increases exponentially as gap size decreases. Huang *et al*. who first introduced DLD designed device to separate 0.6 *μm* fluorescents polystyrene beads with the lateral and downstream gap size of only 1.6 μm^1^. Recent work showed a device with 1.7 μm gap to separate genomic DNA at flow rate of only 0.001 μl/min to 0.01 μl/min^14^.

Even though the downstream and lateral gap size can be tuned to achieve better separation and throughput, there is a breakdown limit for deterministic lateral displacement with different downstream and lateral gap size. This is because the reduction rate in critical diameter is less than the reduction rate of downstream gap, thus there is a point whereby *D*_*c*_ > *G*_*D*_ which means that all particles are displaced for any *θ* while particle smaller than *G*_*D*_ goes in zig-zag mode and DLD become tilted crossflow filtration^20^. This is the case for Davis *et al*. where the WBCs to be separated from blood have larger diameters than *G*_*D*_ and the *G*_*L*_:*G*_*D*_ ratio is in excess of 3 6.

The same limitation also holds for lateral gap variation when the *D*_*c*_ is larger than *G*_*L*_. In this condition, there is no particle separation for any *θ* because the particle larger than the *D*_*c*_ must be larger than the lateral gap and eventually clogs the device, while particle smaller than the *D*_*c*_ travel straight down the device. In this condition, DLD becomes pure 1 dimensional filtration technique.

It is also important to note that the 2 dimensional fluid simulation omits the depth of the device which accounts for G«E, however, in our device the depth is only 11 um which are still in the same order with the 9 um device gap. This could influence the device resistance as the non-slip boundary conditions on the floor and top of the channel also drags the fluids. Our comparison across different DLD devices uses the same height with different gap sizes. Thus though the effects of floor and roof of device can influence the flow resistance, the relative difference on separation can be observed and compared.

The resistance of DLD array is important as high throughput rate is needed for clinical applications such as disease diagnostics. Current technique to increase throughput of DLD device is through stacking the device vertically or in parallel. Inglis *et al*. developed DLD devices with throughput of more than 1 mL/min by integrating six DLD devices in parallel for leucocytes enrichment^21^. Holm *et al*. stacks ten DLD layer vertically to show a high throughput DLD separation device for sleeping sickness detection^22^. This challenge will be exacerbated when the gap sizes are reduced to the sub-micron ranges when even flowing liquid may be challenging due to the exponentially increasing device resistance.

### The effects of G_L_ and G_D_ on particle separation

**Figure 2:**
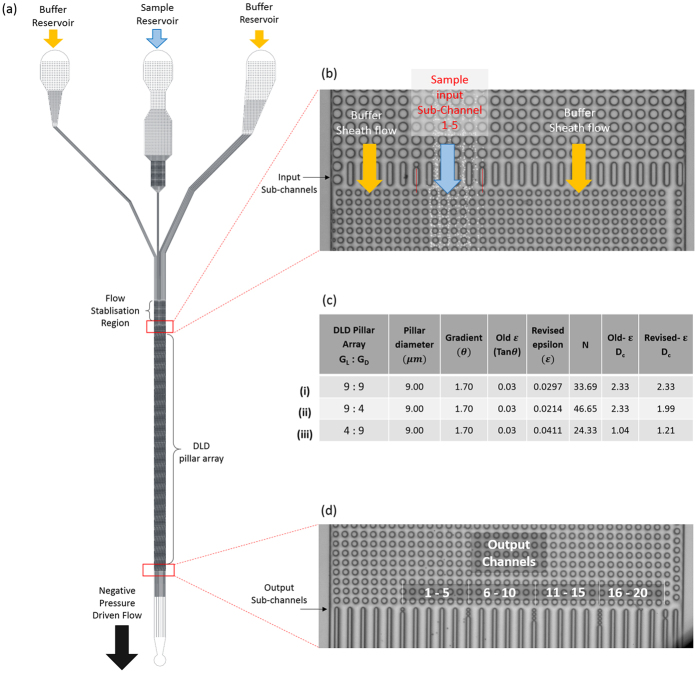
Device schematics and design with different DLD pillar array gap sizes. The overall device schematics are shown in (**a**) where buffer and sample streams are loaded into reservoirs and driven into the device using extraction method by introducing a negative pressure using a syringe pump. As the three fluid input stream converges, a flow stabilization region is required to ensure consistent sample input flow within the sample input region of sub channel 1–5 shown in (**b**). Three DLD devices of different pillar array with gap size ranging from 9 μm:9 μm (i), 4 μm:9 μm (ii) and 9 μm:4 μm (iii). The output region of the device is sub-divided into 22 channels to enable a detailed characterization of the sample separation spectrum.

To test the effects of asymmetrical DLD gap size variation on particle separation, we designed a DLD separating device with three input channels and 22 output sub-channels. Using Equation 1, we designed a conventional DLD device with a gradient of 1.7^o^, symmetrical G_L_ and G_D_ of 9 μm each and pillar diameter of 9 μm. This results in a D_c_ of 2.3 μm. Subsequently, we varied the gap sizes by changing G_L_ and G_D_ to 4 μm respectively (see Fig. 2 and Supplementary Fig. S1). The device has a sample input region at output channels 1 to 5. The sample inputs can be found in Supplementary Fig. S3. Particles can be laterally displaced to the right to a maximum of up to output sub-channel 22. Different sizes of beads were used to characterize the DLD separating device and RBCs were used to test the separation efficiency for irregular biological particle separation. The output composite image of video screen capture and its respective separation spectrum and its separating index (SI) can be seen in Fig. 4 and Supplementary Tables-S3 and S4.

For all three DLD gap-size variations, 2.0 μm beads do not separate from the input sample stream with a SI of less than 50%. For a 9:9 gap setup, 2.5 μm bead slips out of the sample stream at the output channels which shows distinctly poor separation as compared to 4:9 and 9:4 DLD device. A separation is expected for 9:9 as this DLD device has a critical diameter of 2.3 μm. Interestingly, for spherical shaped beads, 4:9 and 9:4 DLD devices outperform the 9:9 DLD device and both have shown similar D_c_ for separation of 2.5 μm beads with SI of 55% and 57% respectively. 9:4 DLD device shows a slight edge over the 4:9 device for bead size 3.0 μm. Based on our revised DLD model (Fig. 2c), both 4:9 and 9:4 should have D_c_ of 1.21 μm and 1.99 μm respectively and 4:9 should have shown a larger edge over bead based particle separation. However, they both show similar separation in beads.

**Figure 3:**
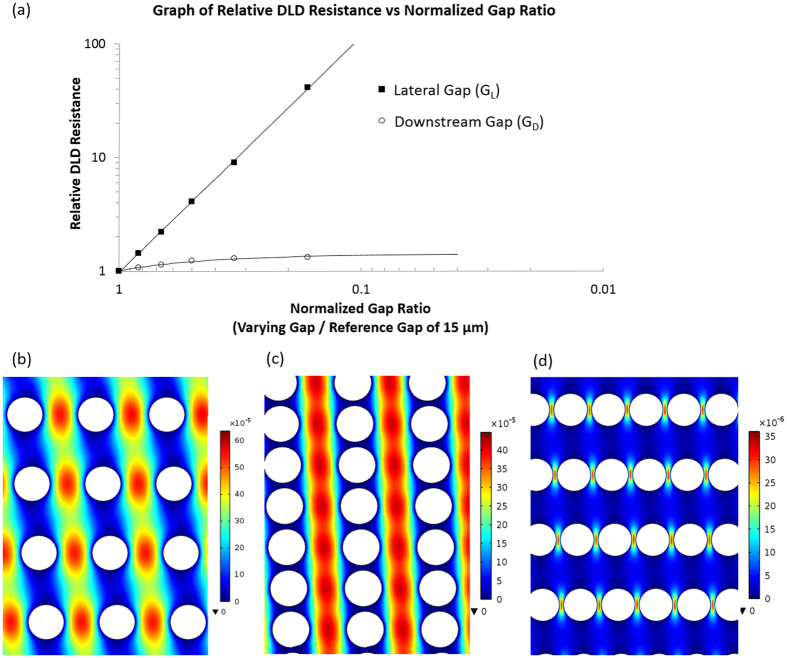
Computational comparison of relative resistance and flow visualization. (**a**) shows the comparison of relative DLD resistance relationship between varying G_L_ and G_D_ gap sizes. The normalized gap ratios and relative resistances were calculated by dividing each gap size and resistance with the reference of symmetric 15:15 gap size and simulated resistance value respectively. Computational flow visualization was performed with individual color scale (m/s) for comparison between G_L_: G_D_ of (**b**) 15:15, (**c**) 15:2 and (**d**) 2:15 DLD arrays.

After a revision of D_c_ (Fig. 2c), this is expected for 9:4 DLD device (from 2.33 μm–1.99 μm) but the revised D_c_ is not so accurate in predicting the separation performance of 4:9 DLD device (from 1.04 μm–1.21 μm). Computational flow model in Fig 3d shows that the flow velocity experienced by a bead flowing between pillars with *G*_*L*_:*G*_*D*_ < 1 change from high velocity between pillar gap to low velocity between rows of pillars. This large velocity gradient could introduce additional fluidic forces not factored in current DLD model which may affect the particle separation. Conversely, the separation data of 9:4 DLD pillars is well predicted by the new revised D_c_ and the effects of anisotropic flow may not be as significant as hypothesized. Especially when Reynold’s number in our experiments are really low (Re ≪ 1). We designed a new DLD device (see Supplementary Figs S4 and S5) with different pillar and gap dimensions and confirmed that the separation is the same when we swap the values of *G*_*L*_:*G*_*D*_ for 15:30 (revised D_c_ = 6.01 μm) and 30:15 (revised D_c_ = 8.15 μm). The separation results for *G*_*L*_:*G*_*D*_ < 1 were accurately predicted but *G*_*L*_:*G*_*D*_ > 1 did not perform as predicted. Thus, the revised D_c_ calculations using Eq. 3. would accurately predict device separations for *G*_*L*_:*G*_*D*_ < 1.

Contrary to spherical beads, disc shape RBCs separation shows a greater sensitivity with a smaller G_D_. We tested RBCs within the DLD devices and found that 9:4 DLD gap-size ratio has a more distinct separation and greater separation selectivity as compared to 4:9 and 9:9. The SI for 9:9, 4:9 and 9:4 are 31.42%, 62.04% and 85.58% respectively. The increase in output separation efficiency is significant (>50%) and even 4:9 gap-size ratios performed better than 9:9 for RBC separation. However, 4:9 DLD devices were very prone to clogging of RBCs (See Supplementary Fig. S6) as the lateral pore is much smaller than the length of the RBC. This result is expected as discussed earlier, the lateral pore influences the resistance and throughput of the device. The narrow lateral pores act as filtration pores and naturally if any particle larger than the pore will not pass through it. Additionally, RBCs are very deformable and as they squeeze between the narrow pores, the added strain and deformation may increase the chances of slippage thus reduced separation efficiency as compared to the 9:4 DLD device (see Supporting Movie 1). When the RBC squeeze through the tiny 4 μm gap, they tend to deform significantly and quite permanently forming a cone shape particle rather than a bi-concaved disc (Fig. 5c and Supplementary Movie 1). The shear stress and deformation are too large even for a flow rate of 0.5 μl/min and would not be a fair comparison since the RBCs shape and deformability have changed. Figure 5 shows the corresponding shear stress for 4:9 DLD device with smaller G_L_ and is much larger than 9:4 and 9:9 DLD devices.

On the contrary, the smaller G_D_ pore for 9:4 DLD device do not hinder the flow of the RBC particles, reduces clogging and additionally increases the separation efficiency of the RBCs while maintaining most of the RBC shape (Fig. 5b). This is because the smaller downstream gap confines the RBCs within the columns of channels as the length of the RBC is much bigger than the G_D_. It is also interesting to note that for 9:4 DLD device, the RBC particle separation mimics a spherical particle of larger than 3.0 μm. However, for 9:9 and 4:9 DLD devices, RBCs all show a diameter of less than 3.0 μm.

While the 11 μm device depth may seem shallow compared to RBC size (8 μm), the RBCs all align the face side to the pillars in all the device except for 4:9. This can be seen in the Supplementary Movie 1. As such, the RBC interaction with the pillar is similar to other works by Holm *et al*. (4 and 10 μm), Beech *et al*. (4 and 10 μm) and our previous work^3^,^4^,^7^,^10^. Although the depth is only 11 um and it might affect the performance of the separation, we could still observe that significant improvement in RBC separation of 9:4 compare to 9:9 device. Our comparison across different devices are using same height with different gap sizes. Thus though the effects of floor and roof of device can influence the flow resistance, the relative difference on separation can be observed.

Reducing G_D_ improves selectivity for separating non-spherical particles like RBCs compared to reducing G_L_. Also, the empirical model seems to breakdown in predicting the effects of asymmetrical gap sizes as the D_c_ cannot be easily predicted. More importantly, current DLD model emphasizes the lateral gap as the dominant factor in determining the critical diameter and we found that this is not true. The downstream gap can also influence the D_c_ and our results have shown that changing downstream gap increases both spherical and non-spherical particle separation efficiency. Our results shed new light to the original DLD model that perhaps the model is not complete. As such, only empirical model based on Equation 1 can be used for symmetric gap DLD.

### Enhancing red blood cells separation with asymmetrical gap DLD

There are several ways to enhance the particle separation. Separation enhancement can be done by coupling DLD with external fields such as dielectrophoresis force to influence the particle separation trajectory^23^. However, coupling DLD with external force fields requires additional equipment to modulate enhancement. Beech and Holms *et al*. propose the use of spatial confinement to allow the RBCs to face flat on the floor of the device^3^,^4^. This increases the size of the RBC from 2 μm to 8 μm, making it easier to separate despite restricting the channel depth to only 4 μm. Our group previously developed a DLD device with I-shaped post array instead of conventional circle shape post array to selectively separate non-spherical particle such as RBCs and rod-shaped Escherichia Coli bacteria^7^. This unique pillar shape induces non-spherical particles to rotate which increase its hydrodynamic radius, thus increasing the separating size^10^.

Our current work of asymmetrical gap sizes also shows comparable separation of RBCs (>90% separation efficiency) as compared to the I-shape pillar. By pushing the limits of G_D_ variation, we developed a DLD device with a gap-size ratio of 9:3 and a device depth of 12 μm and found that it effectively separates the RBCs (see Supplementary Fig. S7). We used a separation index to compare the separation efficiency of both devices and found that while I-shape has a separation index of 100%, the G_D_ gap size variation DLD device had a separation index of >95% as shown in Fig. 6. Separation index (SI) was developed as a means to compare the separation efficiency across various DLD device^10^.

**Figure 4:**
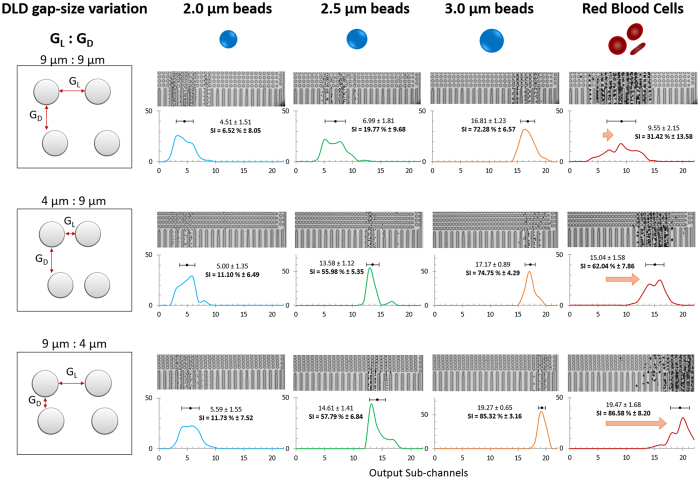
Bead and RBC separation data for asymmetrical DLD gap-size variation. This figure shows three DLD gap-size variation devices (9:9, 4:9 and 9:4) and their corresponding output separation data for 2.0 μm (blue), 2.5 μm (green), 3.0 μm (orange) and RBCs (red). The horizontal axis is the output sub-channels and the vertical axis is the normalized percentage per output sub-channel. The mean sub-channel position and its corresponding separation index (SI) is calculated from the relative input channels shown in Supplementary Fig. S3, Tables S3 and S4. The sample input stream spans in the region of output sub-channels 1 to 5.

**Figure 5:**
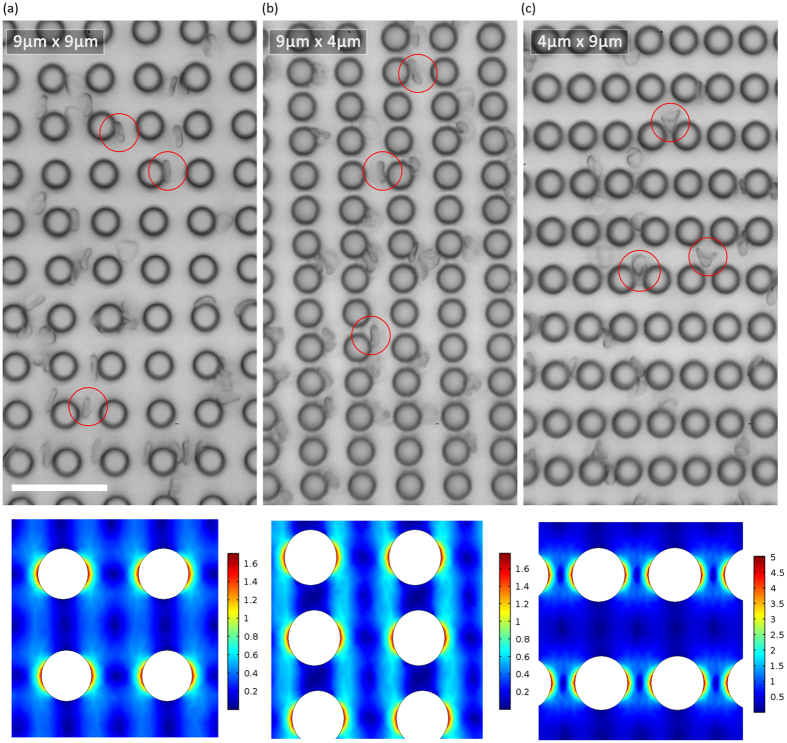
Comparing RBC flow movements within the DLD device. The figure shows a freeze screen capture of RBC flow movement video within a DLD pillar array (Supplementary Video 1). The respective pillar arrangements are shown for various G_L_: G_D_ for (**a**) 9:9, (**b**) 9:4 and (**c**) 4:9 DLD devices. The red circles depict the shape of RBC as it flows within these devices. (**a**,**b**) show more bi-concave disc shaped RBC while (**c**) shows deformed cone shaped RBC. The scale bar is 35 μm. According to the Shear stress simulation of 9:9, 9:4 and 4:9 gaps, it can be observed from the scale that 4:9 has the highest shear stress compare to both 9:9 and 9:4 gaps.

**Figure 6:**
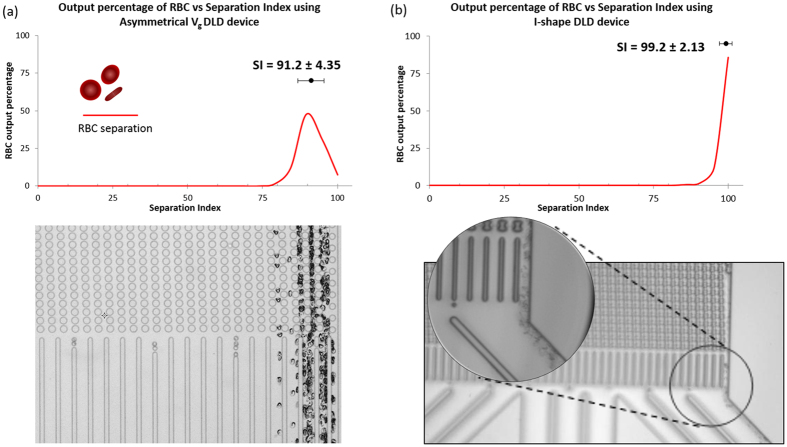
Comparison of RBC separation in asymmetrical DLD gap size and I-shape pillar. (**a**) shows the RBC separation plot based in separation index (SI) for asymmetrical DLD gap size device of G_L_: G_D_ of 9:3. The video composite shows the corresponding separation index. (**b**) shows the separation of RBC using I-shape pillars normalized to SI. This separation data was extracted from our previous work^7^ (Permission have been granted to Authors).

Even though I-shaped device has a larger gap size (10 μm) and is more effective than the G_D_ gap size in controlling RBC separations, its fabrication is much more challenging as compared to the asymmetrical DLD gap device. I-shape device requires precision in fabricating the edged protrusions to trigger rotations and varying fluid flow gradients. The protrusions are of square feature size of 2.5 μm. This fabrication is much more challenging as compare to fabricating a circle pillar feature of 9 μm with a 3 μm downstream gap. Moreover, the D_c_ of the asymmetrical G_D_ DLD device also is enhanced as shown in Fig. 4. The difference lies in both methods employ different separating mechanism. I-shape pillar introduces fluid flow variations to enable non-spherical particles to rotate while asymmetrical G_D_ DLD device simply restricts the cross-flow movement of RBCs by reducing the gap-size significantly relative to the larger RBC.

This method of separating non-spherical RBCs is also much more effective as compared to RBC shape confinement DLD separation method using a shallow 4 μm channel height. Our proposed method is not restricted by the channel height, thus, the throughput of the device is not restricted.

## Conclusion

We have successfully shown how DLD gap size variation can greatly enhance the separation of particles without restricting the flow velocity. It has never been attempted to change downstream gap size of DLD as it was common knowledge that lateral gap influences the critical diameter of particle separation. Here, we found that both lateral and downstream gap size influence the DLD Dc in different ways. More importantly, reducing G_D_ allows little restriction of throughput and greatly enhances separation of non-spherical RBC comparable to previous attempts such as RBC separation using I-shaped pillars. The results of this study suggest that it is possible to design high resolution and throughput deterministic lateral displacement device by tuning the downstream gap in the array design for applications in micron regime separation. This means that the potential application of DLD for particle separation can be fully leveraged by considering both lateral and downstream gap size in the device design process.

## Additional Information

**How to cite this article**: Zeming, K. K. *et al*. Asymmetrical Deterministic Lateral Displacement Gaps for Dual Functions of Enhanced Separation and Throughput of Red Blood Cells. *Sci. Rep*. **6**, 22934; doi: 10.1038/srep22934 (2016).

## Supplementary Material

Supplementary Information

Supplementary Movie S1
