# Supplementary material for: Asymmetrical Deterministic Lateral Displacement Gaps for Dual Functions of Enhanced Separation and Throughput of Red Blood Cells
Source: Sci Rep. 2016 Mar 10;6:22934. doi: 10.1038/srep22934 (PMC4785430; doi:10.1038/srep22934)
Supplement: Supplementary Information [file srep22934-s1.pdf]

ASYMMETRICAL DETERMINISTIC LATERAL DISPLACEMENT GAPS FOR DUAL FUNCTIONS OF ENHANCED SEPARATION AND THROUGHPUT OF RED BLOOD CELLS

Kerwin Kwek Zeming[a,b,c#], Thoriq Salafi[a,b,d#], Chia-Hung Chen[a,c], Yong Zhang[a,b,d]*

Author affiliation:

a: Department of Biomedical Engineering, National University of Singapore, 4 Engineering Drive 3, Block E4 #04-08, Singapore 117583

b: Cellular and Molecular Bioengineering Lab, National University of Singapore, Block E3A, #07-06, 7 Engineering Drive 1, Singapore 117574

c: Singapore Institute for Neurotechnology (SINAPSE), 28 Medical Dr. #05-COR, Singapore 117456

d: NUS Graduate School for Integrative Sciences and Engineering, Centre for Life Sciences (CeLS), National University of Singapore, 05-01 28 Medical Drive, Singapore 117456, Singapore

#Co-first authors

*Corresponding Author:

Yong Zhang
Department of Biomedical Engineering,
National University of Singapore,
4 Engineering Drive 3, Block E4 #04-08, Singapore 117583
+65 6516 4871
biezy@nus.edu.sg

Keywords

Deterministic lateral displacement, Red Blood Cell, Dual Gap

Supplementary Information 1: The revised D_c value of different downstream and lateral gap from the revised ε formula

Simulation parameter	Value
**Software**	COMSOL Multiphysics v4.4
Physics type (In COMSOL)	Creeping flow (stokes flow)
Simulation type (steady or transient)	Steady
Main solver	Fully coupled
Linear solver	Iterative
Mesh Element	Triangular and quadrilateral element type
**Geometry**	
Pillar size (D_p)	15 μm
Gap size (H_g:V_g)	15:2.5, 15:5, 15:7.5, 15:10, 15:12.5,<br>2.5:15, 5:15, 7.5:15, 10:15, 12.5:15, and 15:15
Row shift fraction (ε)	0.0287 - 0.0844 (depends on the G_L, G_D, θ, D_p)
Width (W)	150 μm
Length (L)	320 μm
**Flow properties**	
Material	Water
Viscosity (μ)	0.001 Pa.s
Density (ρ)	$1000 kg/m^3$
Reynold number (Re)	$1.31 * 10^{-5}$ to $3.26 * 10^{-5}$
**Boundary Condition**	
Pressure Difference (ΔP)	15 Pa
Walls	No-slip condition

Supplementary Table-S1: COMSOL parameters for DLD resistance and throughput computational modeling.

Supplementary Information 2: The device specifications and calculations

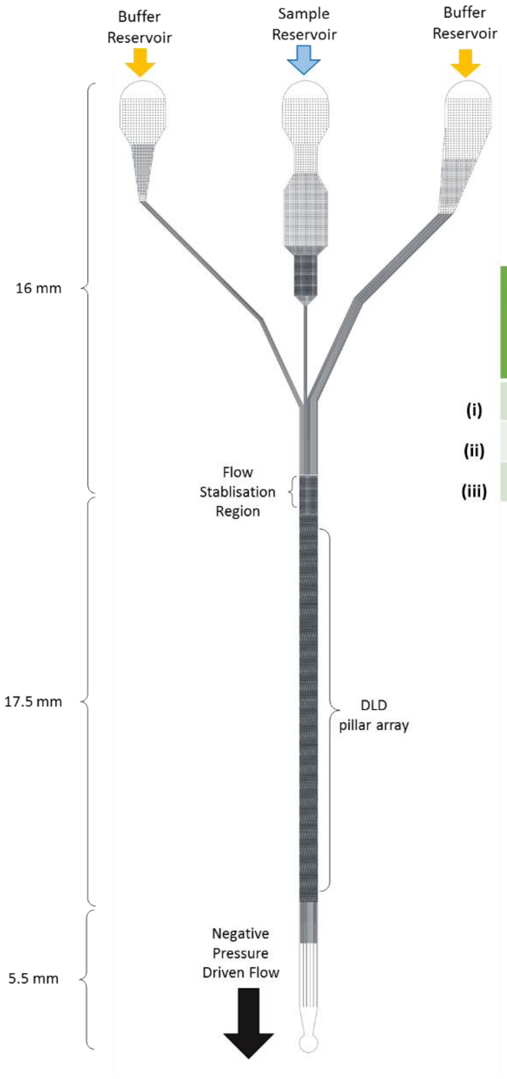

	DLD Pillar Array $G_L : G_D$	Pillar diameter (μm)	Lateral period $(\lambda_L, \mu m)$	Downstream period $(\lambda_D, \mu m)$	Gradient (θ)	Old ε (Tanθ)	Old D_c	Revised ε	Revised D_c
(i)	**9 : 9**	9.00	18.00	18.00	1.70	0.03	**2.33**	0.0297	**2.33**
(ii)	**9 : 4**	9.00	18.00	13.00	1.70	0.03	**2.33**	0.0214	**1.99**
(iii)	**4 : 9**	9.00	13.00	18.00	1.70	0.03	**1.04**	0.0411	**1.21**

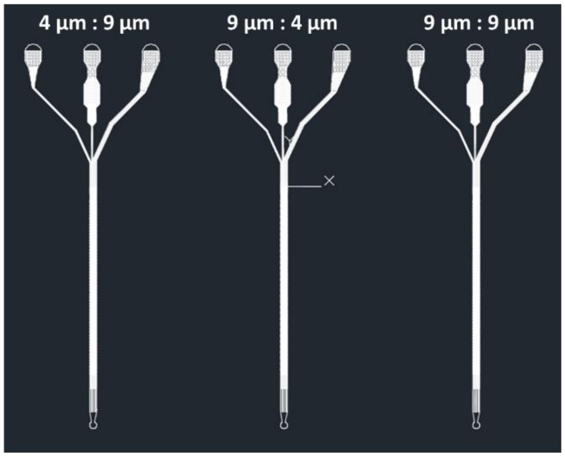

Supplementary Figure-S1: Device dimensions and specifications in the mask design. The dimensions of the DLD device is approximately 39 mm. Three DLD devices were made with differing gap sizes of 9:9, 9:4 and 4:9 respectively. The mask designs are shown in the figure.

Supplementary Information 3: The revised D_c value of different downstream and lateral gap from the revised ε formula

$$\lambda_L = G_L + D_p$$

$$\lambda_d = G_D + D_p$$

$$Old\ \varepsilon = tan\ \theta$$

$$Revised\ \varepsilon\ = \frac{(G_D + D_p)tan\theta}{G_L + D_p}$$

Pillar diameter $(D_p, \mu m)$	Lateral gap $(G_L, \mu m)$	Lateral period $(\lambda_L, \mu m)$	Downstream gap$(G_D, \mu m)$	Downstream period $(\lambda_D, \mu m)$	Gradient (θ)	Old ε (Tanθ)	Old D_c	Revised ε	Revised D_c
15.00	**15.00**	30.00	**15.00**	30.00	2.82	0.05	**4.95**	0.0493	**4.95**
15.00	**15.00**	30.00	**12.50**	27.50	2.82	0.05	**4.95**	0.0452	**4.75**
15.00	**15.00**	30.00	**10.00**	25.00	2.82	0.05	**4.95**	0.0410	**4.54**
15.00	**15.00**	30.00	**7.50**	22.50	2.82	0.05	**4.95**	0.0369	**4.31**
15.00	**15.00**	30.00	**5.00**	20.00	2.82	0.05	**4.95**	0.0328	**4.07**
15.00	**15.00**	30.00	**2.50**	17.50	2.82	0.05	**4.95**	0.0287	**3.82**
15.00	**15.00**	30.00	**15.00**	30.00	2.82	0.05	**4.95**	0.0493	**4.95**
15.00	**12.50**	27.50	**15.00**	30.00	2.82	0.05	**4.13**	0.0537	**4.30**
15.00	**10.00**	25.00	**15.00**	30.00	2.82	0.05	**3.30**	0.0591	**3.60**
15.00	**7.50**	22.50	**15.00**	30.00	2.82	0.05	**2.48**	0.0657	**2.84**
15.00	**5.00**	20.00	**15.00**	30.00	2.82	0.05	**1.65**	0.0739	**2.00**
15.00	**2.50**	17.50	**15.00**	30.00	2.82	0.05	**0.83**	0.0844	**1.07**

Supplementary Table-S2: old epsilon and revised epsilon calculation for asymmetric gap DLD with the old and revised D_c calculation. In the simulation, Pillar diameter of 15μm and gradient of 2.82 degree are used. we fixed lateral gap of 15μm, and varied the downstream gap from 2.5 to 15μm and subsequently varied the lateral gap from 2.5 to 15 μm while the downstream gap is fixed at 15μm. We can see that the old ε values are all the same as the gradient values are equal. However, in revised ε, the values of row shift fractions are different as it takes into account of both downstream gap and lateral gap. It can be observed that the revised epsilon values go smaller as the downstream gap sizes are reduced compare to the higher epsilon values when the lateral gap sizes are reduced. In the revised epsilon, pillar diameter influences the row shift fraction as the larger pillar diameter requires longer distance for a pillar to be shifted by one period.

To calculate the revised Dc, the revised ε is used for the equation $D_c = 1.4g\varepsilon^{0.48}$ where g = lateral gap. This lateral gap g is the same as G_L. Compared to the old Dc, DLD parameters with $G_L{:}G_D > 1$ showed enhancement while the old Dc would remain unchanged no matter how small G_D gets. This was the case for Davis et al where using the old formula regardless of G_D would result in an inaccurate expectations or "overkill" DLD separation parameters for particle separation.[1] On the contrary, $G_L{:}G_D < 1$ showed much better

enhancements to Dc as compared to G_L: $G_D > 1$. This was counter intuitive and needed to be thoroughly investigated.

Supplementary Information 4

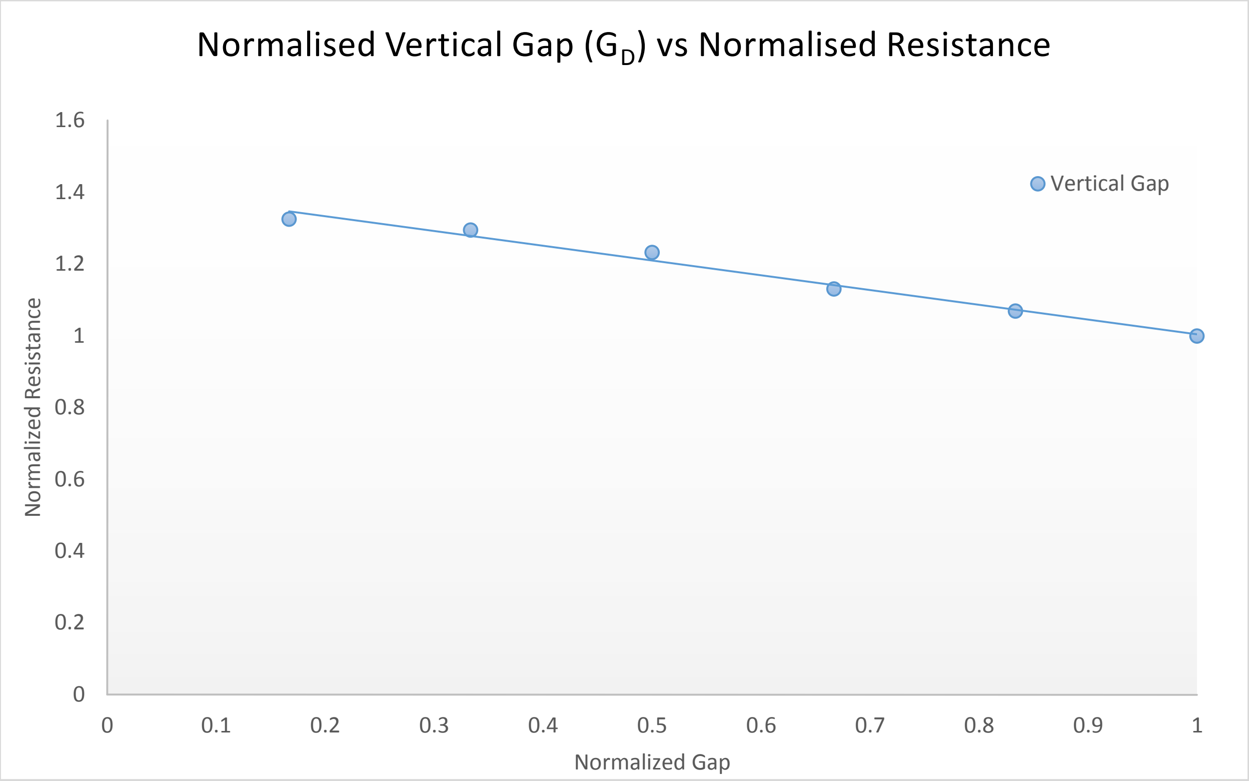

Supplementary Figure-S2: Graph of normalized vertical gap (G_D) vs normalized resistance.

For cases where $G_L : G_D > 1$ such that G_D is smaller than G_L, the resistance increases linearly as G_D becomes smaller. The gradient is negative. For $G_L : G_D < 1$, the resistance increases exponentially when the lateral gap decreases.

Supplementary Information 5: Sample input distribution for various DLD devices.

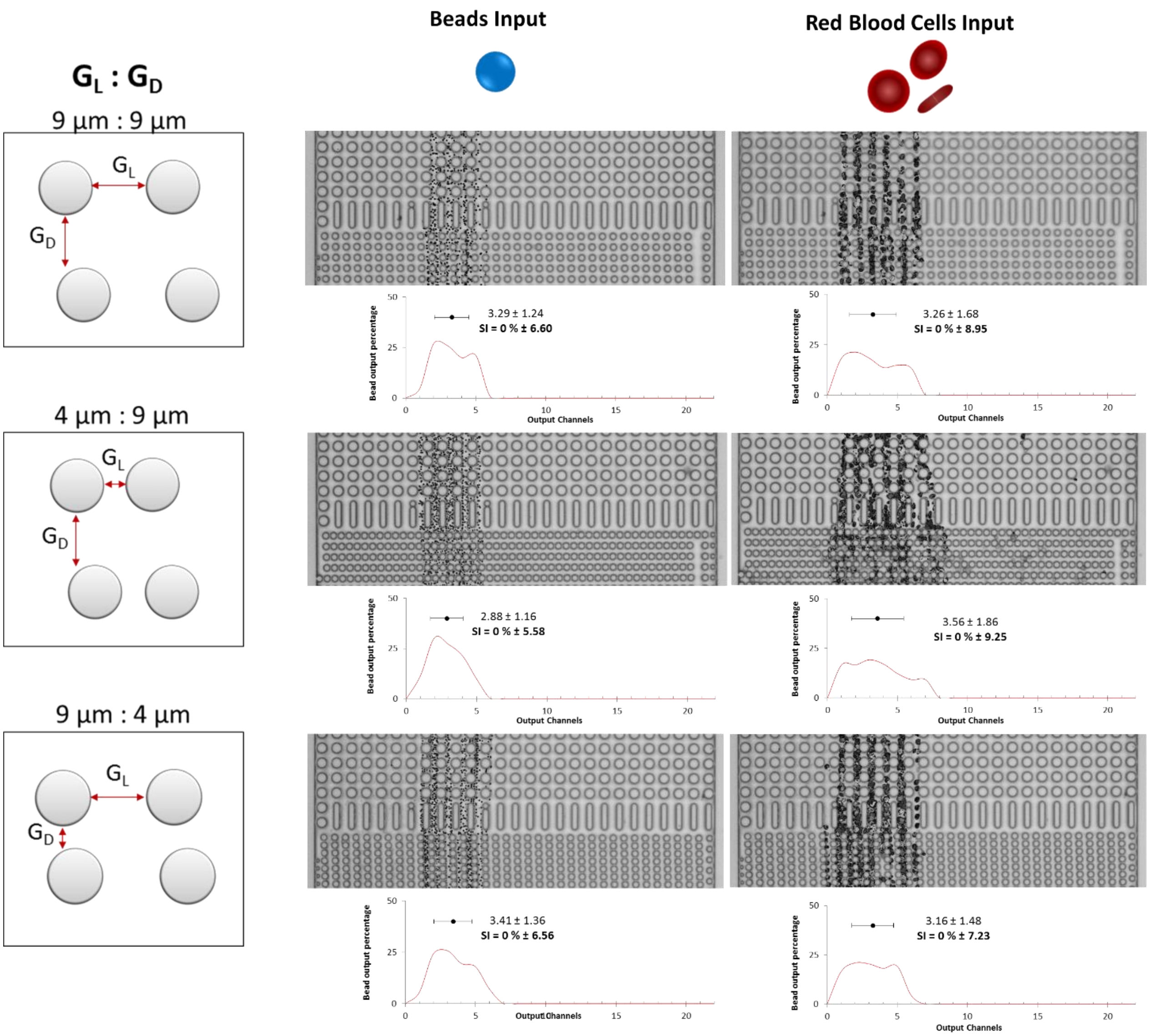

Supplementary Figure-S3: Input sample spectrums for beads and RBCs. The figure shows the input separation of beads and RBC for *9µm : 9µm , 4µm : 9µm and 9µm : 4µm.*

Supplementary Information 6: Calculations of sample mean position and Separation Index (SI)

We have additionally added the data analysis of the mean and standard deviation of the spectrum based on mean sub-channel position and corresponding SI values.

	**Input and Output Sub-channel Mean Position**					
	**9 x 9 Array**	**SD**	**4 x 9 Array**	**SD**	**9 x 4 Array**	**SD**
**Input**	**3.29**	1.24	**2.88**	1.16	**3.41**	1.36
**1.5 µm**	**4.38**	1.40	**4.48**	1.09	**4.91**	1.36
**2.0 µm**	**4.51**	1.51	**5.00**	1.35	**5.59**	1.55
**2.5 µm**	**6.99**	1.81	**13.58**	1.12	**14.16**	1.41
**3.0 µm**	**16.81**	1.23	**17.17**	0.89	**19.27**	0.65
**RBC input**	**3.26**	1.68	**3.56**	1.86	**3.16**	1.48
**RBC output**	**9.15**	2.55	**15.04**	1.58	**19.47**	1.68

Supplementary Table-S3: Tabulating the mean separating input and output sub-channel position for the respective samples of beads and RBC.

We have included 1.5 micron beads in this data to show that particle size smaller than 2.0 microns have similar separation spectrum as 2.0 microns.

	**Separation Index (%)**					
	**9 x 9 Array**	**SD**	**4 x 9 Array**	**SD**	**9 x 4 Array**	**SD**
**Input**	**0.00**	6.60	**0.00**	5.58	**0.00**	6.56
**1.5 µm**	**5.80**	7.49	**8.39**	5.23	**8.05**	6.60
**2.0 µm**	**6.52**	8.07	**11.10**	6.49	**11.73**	7.52
**2.5 µm**	**19.77**	9.68	**55.98**	5.35	**57.79**	6.84
**3.0 µm**	**72.28**	6.57	**74.75**	4.29	**85.32**	3.16
**RBC input**	**0.00**	8.95	**0.00**	9.25	**0.00**	7.23
**RBC output**	**31.42**	13.58	**62.24**	7.86	**86.58**	8.20

Supplementary Table-S4: Coressponding SI values of the separation.

In order to compare the various separation results between pillars, a separation index is used. The strength and quality of separation is expressed in the magnitude of the index while the resolution is denoted in the standard deviation. The advantage of using an index is to have a standard method of comparison between various devices across all DLD experiments regardless of the number or length at output positions.

The formula for the index is as follow:

$$\frac{Sample\ Displacement - Initial\ Sample\ Position}{Max\ Sample\ Displacement - Initial\ Sample\ Position} \times 100$$

Separation Index (SI) = $\frac{\overline{X} - X_{Input\ Mean}}{22 - X_{Input\ Mean}} \times 100$ --------- (Supplmentary Eq. 1)

$\overline{X}$ = the mean deviation of the sample stream as depicted in the graphs.

Supplementary Information 7: Secondary DLD experiment to test the separation of 15:30 and 30:15 DLD devices.

The device were fabricated using standard lithography methods of negative photo resist in SU-8 mold. A photomask was sent for printing and used to pattern SU-8 2025 on a 4” silicon wafer. The SU-8 2025 was spin at 3500 rpm and soft backed at 95°C for 10 mins. The resulting thickness of the SU-8 mold on the wafer is approximately 20 µm thick. The mask was attached to a mask aligner and the wafer was exposed to 365nm UV light for a total energy exposure of 140 mJ/cm^2. The post-exposure back was performed at 95°C for 2 mins and the patterns were developed under the SU-8 developer wash for 5 mins. The mask designs are as follow:

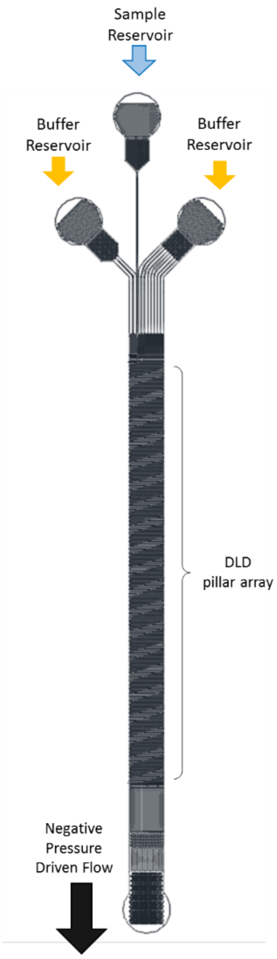

Pillar diameter ($D_p, \mu m$)	Lateral gap ($G_L, \mu m$)	Lateral period ($\lambda_L, \mu m$)	Downstream gap ($G_D, \mu m$)	Downstream period ($\lambda_D, \mu m$)	Gradient (θ)	Old ε (Tanθ)	Old D_c	Revised ε	Revised D_c
**15.00**	15.00	30.00	30.00	45.00	2.82	0.05	4.95	0.0739	**6.01**
**15.00**	30.00	45.00	15.00	30.00	2.82	0.05	9.90	0.0328	**8.15**

Supplementary Figure-S4: Mask design of a 15:30 and 30:15 DLD device.

After the SU-8 mold is complete, we pour a PDMS mixture of 1:10 curing agent to polymer agent into the SU-8 mold, degas the device and let the PDMS cure at 75 °C for 1 hour. Three reservoir holes and a tubing

insert hole were punched at the inlet and outlet respectively. The PDMS device was bonded into a glass slide using oxygen plasma surface activation. The once the device is ready, the same priming and surface treatment techniques were used for these devices.

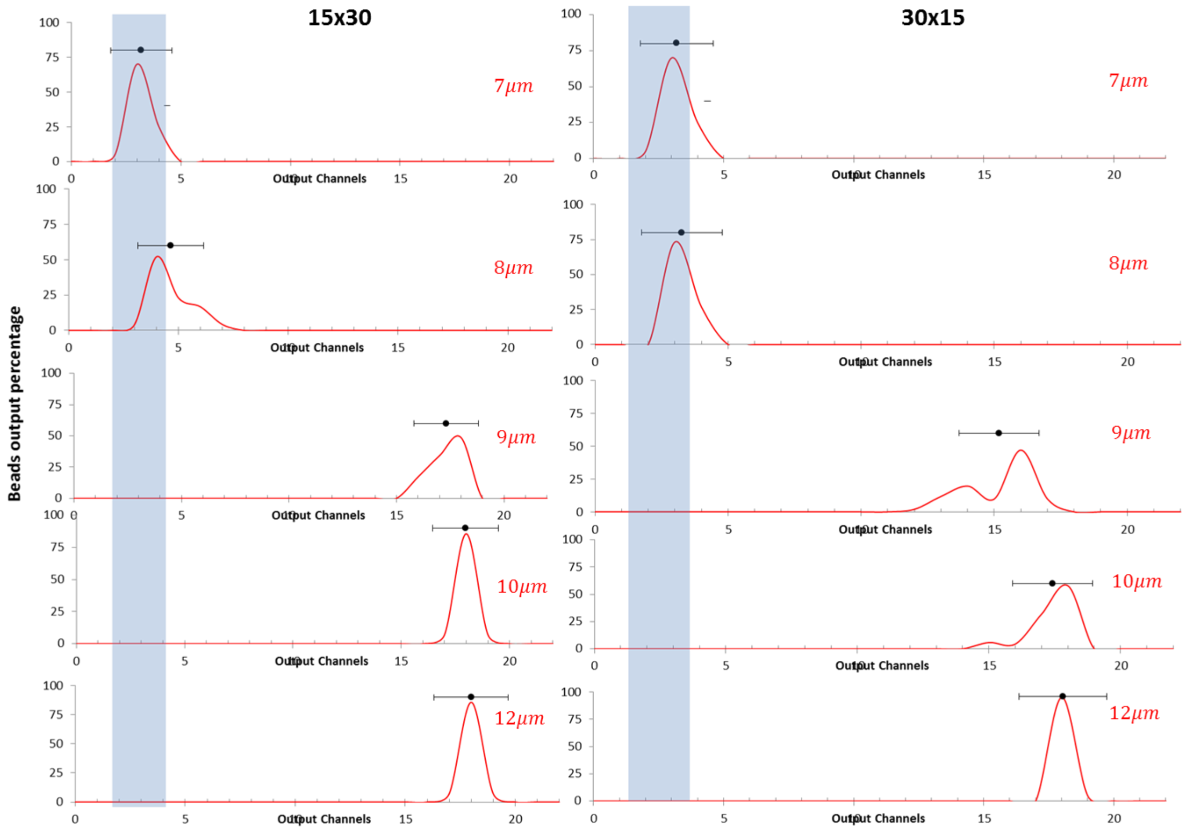

Supplementary Figure-S5: comparison of Dc characterization between 15x30 and 30x15 gap size. We characterized the device of 15x30 and 30x15 with gradient of 2.82° by flowing polystyrene beads of 7, 8, 9, 10, and 12µm in size using pluronic 1% buffer with shaded blue region as inlet positions. It can be seen from figure above that the critical diameter of both devices are similar as beads particle equal and larger than 9µm beads are started to go in bumping mode. This means that the downstream gap also contributes to the critical diameter of the device which is not reflected on DLD empirical formula.

Due to the device being bigger, a larger 1 ml syringe is used and the flow rate could be easily increased to 1 µl/ml. The separation spectrum of the devices are shown here in Figure-S3 which was captured using the highspeed camera and tabulated. The results showed great similarity to separation results of 4:9 and 9:4 DLD devices. They have the same Dc and similar separation efficiency and the revised Dc accurately predict the separation in the 30:15 device (D_c = 8.15 µm). Particles less than 8.15µm cannot be separated in the device.

Supplementary Information 8: DLD devices were very prone to clogging of RBCs

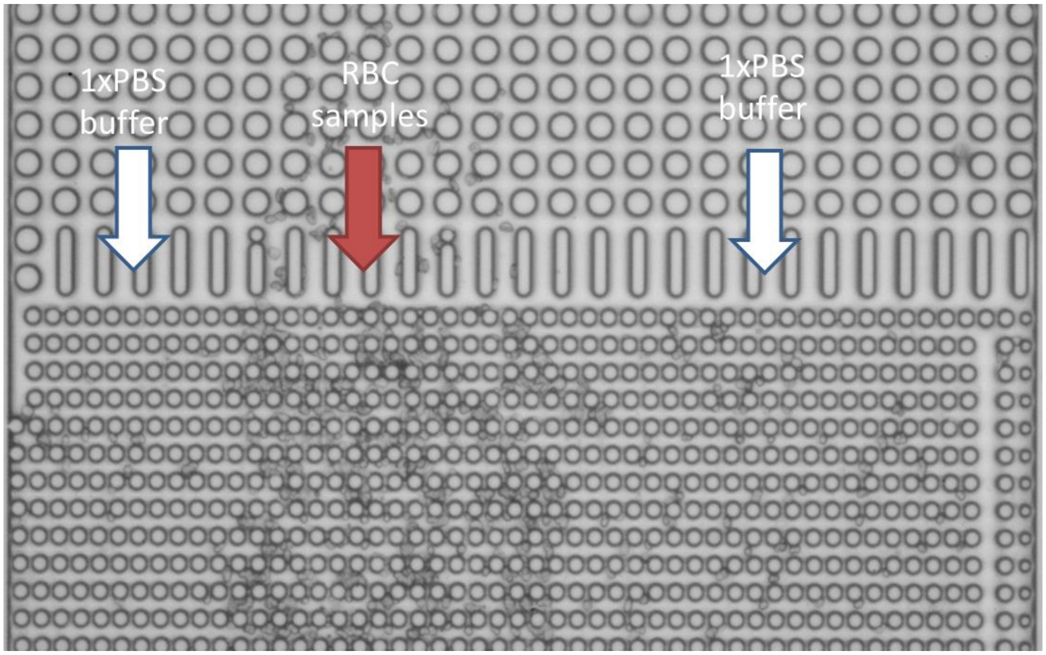

Supplementary Figure-S6: Clogged device input region of the 4:9 DLD device.

The figure shows two 1x PBS buffer solution sandwiching the RBC sample stream of region 1 to 5 marked by the two dots. Due to the clogging, some of the RBC sample stream overflow into the other input sub-channels. Because the RBCs are much larger than the gap size, they tend to clog even if most cells can squeeze through. Once a pore is blocked, there is increase chance of aggregation of more RBCs.

Supplementary Information 9: Fabrication and Gap Size calculations

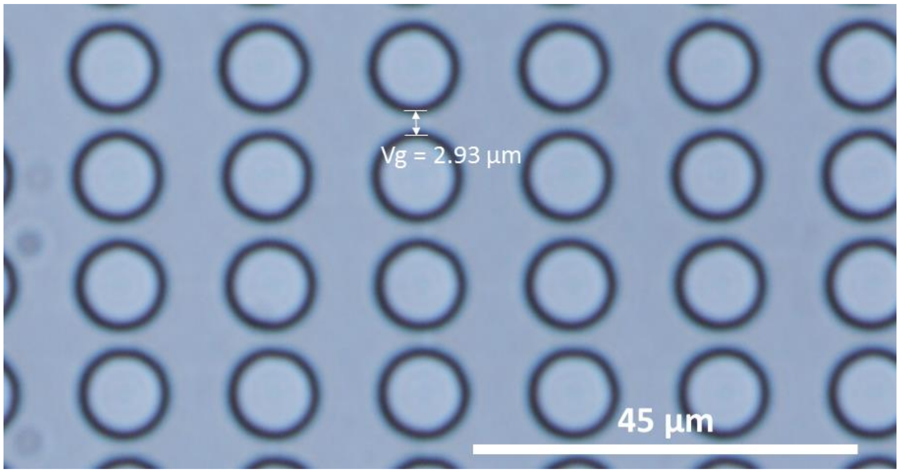

Supplementary Figure-S7: Gap size calculation for PDMS 9:3.

The G_D was reduced to approximately 3µm. The PDMS device was fabricated using an SU-8 mold. The SU-8 mold was made using the same photo mask. The fabrication techniques used were the same as Supplementary Information 7 except that the SU-8 used was SU-8 2010 and the SU-8 was spun at 2500 rpm which resulted in approximately 12 µm. This device has the same design except that it has a smaller gap size due to shrinkage of the PDMS.

Supplementary Movie 1: Comparing the flow movements of RBC in different DLD devices of 9µm : 9µm, 9µm : 4 µm and 4µm : 9µm.

The flow movements were captures at different frame rates but sync together to allow ease of viewing.

References:

1 Davis, J. A. *et al.* Deterministic hydrodynamics: taking blood apart. *Proceedings of the National Academy of Sciences of the United States of America* **103**, 14779-14784.
